# Supplementary material for: The Systems Biology Research Tool: evolvable open-source software
Source: BMC Syst Biol. 2008 Jun 29;2:55. doi: 10.1186/1752-0509-2-55 (PMC2446383; doi:10.1186/1752-0509-2-55)
Supplement: Additional file 1 — SBRT Archive. An archive of the current version of the Systems Biology Research Tool. [file 1752-0509-2-55-S1.zip › sbrt-1.4.0/doc/developers_guide/api/sbrt/kernel/math/graph_theory/class-use/Path.html]

Uses of Class sbrt.kernel.math.graph\_theory.Path


|  |  |  |
| --- | --- | --- |
| **Overview** \| **Package** \| **Class** \| **Use** \| **Tree** \| **Deprecated** \| **Index** \| **Help** | |  |
| PREV   NEXT | **FRAMES**    **NO FRAMES**     **All Classes** |  |


---


## **Uses of Class sbrt.kernel.math.graph\_theory.Path**

| Packages that use Path | |
| --- | --- |
| **sbrt.kernel.math.graph\_theory.comp** | Provides classes and interfaces for performing graph theoretical computations. |
| **sbrt.kernel.math.graph\_theory.proc** | Provides classes and interfaces for performing graph theoretical processes. |

Uses of Path in sbrt.kernel.math.graph\_theory.comp

| Methods in sbrt.kernel.math.graph\_theory.comp that return types with arguments of type Path | |
| --- | --- |
| `java.util.List<Path<N>>` | `PathFinder.getPaths()`             Returns a list of all paths. |
| `java.util.List<Path<N>>` | `PathFinder.getPaths(java.util.Collection<? extends N> sources, java.util.Collection<? extends N> sinks)`             Returns a list of all paths between the provided source and sink nodes. |
| `<T extends N,S extends N> java.util.List<Path<N>>` | `PathFinder.getPaths(T source, S sink)`             Returns a list of all paths between the provided source and sink nodes. |

Uses of Path in sbrt.kernel.math.graph\_theory.proc

| Method parameters in sbrt.kernel.math.graph\_theory.proc with type arguments of type Path | |
| --- | --- |
| `void` | `CycleIdProcess.run(ResultsManager<Path<N>> resultsManager)`             Adds to the provided results manager each identified cycle in the current graph. |
| `void` | `PathIdProcess.run(ResultsManager<Path<N>> resultsManager)`             Adds to the provided results manager each computed path in the current graph. |

---


|  |  |  |
| --- | --- | --- |
| **Overview** \| **Package** \| **Class** \| **Use** \| **Tree** \| **Deprecated** \| **Index** \| **Help** | |  |
| PREV   NEXT | **FRAMES**    **NO FRAMES**     **All Classes** |  |


---
